# Decellularised Cartilage ECM Culture Coatings Drive Rapid and Robust Chondrogenic Differentiation of Human Periosteal Cells

**DOI:** 10.3390/bioengineering9050203

**Published:** 2022-05-10

**Authors:** Wollis J. Vas, Mittal Shah, Helen C. Roberts, Scott J. Roberts

**Affiliations:** 1 Department of Materials and Tissue, Institute of Orthopaedics and Musculoskeletal Science, University College London, Stanmore HA7 4LP, UK; wollis.vas@gmail.com (W.J.V.); mittal.shah@ucl.ac.uk (M.S.); 2 Department of Natural Sciences, Faculty of Science & Technology, Middlesex University, London NW4 4BT, UK; h.roberts@mdx.ac.uk; 3 Department of Comparative Biomedical Sciences, The Royal Veterinary College, London NW1 0TU, UK

**Keywords:** decellularised, extracellular matrix, chondrogenic differentiation, periosteum-derived cell

## Abstract

The control of cell behaviour in an effort to create highly homogeneous cultures is becoming an area of intense research, both to elucidate fundamental biology and for regenerative applications. The extracellular matrix (ECM) controls many cellular processes in vivo, and as such is a rich source of cues that may be translated in vitro. Herein, we describe the creation of cell culture coatings from porcine decellularised hyaline cartilage through enzymatic digestion. Surprisingly, heat-mediated sterilisation created a coating with the capacity to rapidly and robustly induce chondrogenic differentiation of human periosteal cells. This differentiation was validated through the alteration of cell phenotype from a fibroblastic to a cuboidal/cobblestone chondrocyte-like appearance. Moreover, chondrogenic gene expression further supported this observation, where cells cultured on heat sterilised ECM-coated plastic displayed higher expression of *COL2A1*, *ACAN* and *PRG4* (*p* < 0.05) compared to non-coated plastic cultures. Interestingly, *COL2A1* and *ACAN* expression in this context were sensitive to initial cell density; however, *SOX9* expression appeared to be mainly driven by the coating independent of seeding density. The creation of a highly chondrogenic coating may provide a cost-effective solution for the differentiation and/or expansion of human chondrocytes aimed towards cartilage repair strategies.

## 1. Introduction

It is widely accepted that disparity between the physiological cell microenvironment and in vitro conditions cause phenotypic changes that can result in the loss of native function [1], which can subsequently alter cellular response to exogenously added growth factors or drugs [2,3]. An example of this occurs when chondrocytes are cultured on tissue culture plastic. Over as little as two passages, they rapidly lose their phenotype in a process known as dedifferentiation [4]. This limited capacity for in vitro expansion restricts their therapeutic use to procedures dealing with small cartilage defects using autologous chondrocyte implantation (ACI). Several groups have, therefore, suggested the use of chondroprogenitors as an alternative for cartilage tissue engineering [5,6,7]. This approach, however, requires consideration of optimal conditions for their chondrogenic differentiation. Amongst the various techniques that have been investigated to optimise chondrogenesis, the control of surface topology appears to be a promising approach. Indeed, a study by Wu et al. reported the modulation of nano-topographical design using thermal nanoimprinting on polycaprolactone surface to generate various nano-patterns. These were assessed for their effect on cell phenotype and differentiation [8]. The investigation revealed that changes in the surface nanotopography directly affected cellular cytoskeleton arrangement, resulting in morphological changes. Moreover, they also found that specific patterns resulted in enhanced chondrogenesis and cartilage-specific ECM deposition when compared to a non-patterned surface. This is supported by previous studies that establish the link between a more rounded spherical shape and chondrogenic phenotype [9,10].

Beyond synthetic/artificial topographical modulation of cell behaviour, the role of the extracellular matrix (ECM) in regulating cell survival, attachment, proliferation and differentiation is well documented [11,12]. However, most studies primarily investigate single ECM associated proteins as an in vitro cell culture coating, and subsequently define their influence on cell behaviour [13,14]. Some strategies attempt to mimic the more complex native microenvironment using a combination of ECM-derived components, such as fibronectin, collagen and laminin [15,16], with varying levels of success. With relevance to chondrogenesis, the use of cartilage-ECM components to induce differentiation in vitro has been studied, which highlighted their advantages over synthetic biomaterials [17,18]. Indeed, Cheng and colleagues show that recombinant human fibrillin-1 and fibronectin fragments are capable of driving chondrogenesis of human embryonic stem cells. There is also further evidence to suggest enhanced chondrogenesis in cultures that employ collagen type 2 [19,20], which is a key component of hyaline cartilage ECM. Lei and colleagues further strengthen the concept of using cartilage-associated ECM components by demonstrating enhanced chondrogenesis in cultures that include heparin sulfate [21]. The addition of individual ECM-associated components, however, does not mimic the complex tissue-specific combinations, or ratios present in the cartilage ECM. To address this, research has been conducted on cell-derived ECM coatings; an approach has also been harnessed to produce commercially available gel coatings such as Matrigel. The inclusion of these cell-derived culture coatings has been shown to enhance cell attachment and viability [22]; however, they do not mimic tissue-specific ECM such as that found in hyaline cartilage. As such, it would be a major step forward to develop a culture coating that is target cell type/tissue ECM-derived, an approach that has been employed by DeQuach et al. to direct muscle cell differentiation [23]. Despite the overwhelming evidence that natural ECM and components are beneficial for the differentiation of various cell types, there is no study that aims to develop a chondroinductive cartilage ECM-derived tissue culture coating.

## 2. Materials and Methods

### 2.1. Costal Cartilage Harvest

Fresh porcine costal cartilage was obtained from Large-White/Landrace crossbred pigs ranging from 40 to 70 Kgs (being terminated from unrelated studies at Northwick Park Institute for Medical Research; NPIMR, London, UK; approximately 8 ribs were harvested from each pig; *n* = 5). Lateral thoracic incisions allowed access to the costal regions of the rib cage. Costal cartilage directly adjacent to the bone tissue was isolated from the rib cage. Any remaining adherent soft tissue was removed using a sterile scalpel and the harvested costal cartilage placed in sterile plastic bags and stored immediately at −20 °C.

### 2.2. Decellularisation and Digestion

Frozen costal cartilage samples were defrosted at room temperature. The cartilage tissue was dissected into discs measuring 10 mm in diameter and with a volume of approximately 785 mm^3^. Decellularisation was carried out under negative pressure at 2000 mmHg (267 Pa), using a negative pressure desiccator (Sigma-Aldrich, Dorset, UK), using the vacuum-assisted osmotic shock methodology as described in [24]. The digestion methodology was adapted from Sawkins and colleagues [25]. Briefly, the digestion solution was prepared by first adding 50 µL of a 10 M HCL solution to deionised water, and then mixed gently. Next, 50 mg of pepsin (Sigma-Aldrich, Dorset, UK) was added to the solution, giving a final pepsin concentration of 1 mg/mL in 0.01 M HCL. Sterile freeze-milled (Spex sample prep, London, UK) decellularised costal cartilage extracellular matrix (dcECM) granules were added into the digestion solution at a concentration of 10 mg/mL and mixed thoroughly to ensure no clumps were present. The digestion mix was left to digest for 96 h at 21 °C. Digests were stored at 4 °C and subsequently sterilised prior to being used to coat tissue culture plastic.

### 2.3. Sterilisation and Coating of dcECM-Derived Digests

The dcECM-derived digest was sterilised using either UV light or heat-mediated autoclave sterilisation. For UV sterilisation, 30 µL of the dcECM digest was pipetted onto each well of a 96-well plate. Samples were left under UV light in a tissue culture flow hood (Thermofisher, Dartford, UK) for 12 h at room temperature (18–21 °C); the digest coating was further dried without UV exposure for 60 h. Autoclave sterilisation was carried out by pipetting 4 mL of the dcECM digest into glass autoclave tubes (Corning, Ewloe, UK). Samples were subsequently autoclaved (Invitrogen, Renfrew, UK) using the standard media sterilisation cycle. Then, 30 µL of the solution was pipetted onto 96-well plates and left to dry in a sterile dry incubator (Thermofisher, Dartford, UK) for 72 h, after which cells were seeded onto the surface. As a control, dcECM-derived digest was coated as previously described, without any sterilisation steps, and referred to as non-sterile (NS) coatings.

### 2.4. Human Periosteum-Derived Cell (hPDC) Culture

The hPDCs are a key cell skeletal cell population, playing a major role in both bone homeostasis and fracture repair [26] and are, therefore, an ideal cell type for investigating the in vitro bioactivity of the dcECM. The hPDCs were isolated as previously described (obtained from Prof Frank Luyten, KU Leuven, Belgium). All experiments described herein were performed with pooled/banked hPDCs at passage 6 (continuous line). All cell counting was carried out by combining 0.4% trypan blue and culture media at a ratio of 1:2, that was manually counted using a Neubauer hemocytometer (Sigma-Aldrich, Dorset, UK). The hPDCs were seeded onto the surface of the dcECM-derived digest coated wells (96-well plates) to assess cell attachment and differentiation. Control cultures of the same density (*n* = 3) were seeded onto the surface of the 96-well plate as a control. Cells were left to attach for 3 h before the media was changed to CTRL or chondrogenic C(+) media (low-glucose) DMEM (Gibco; Thermofisher, Dartford, UK) supplemented with 100 µM ascorbate-2-phosphate (Sigma-Aldrich, Dorset, UK), 100 nM dexamethasone (Sigma-Aldrich, Dorset, UK), 40 mg/mL proline (Sigma-Aldrich, Dorset, UK), ITS+ premix universal culture supplement (BD Biosciences, Bedford, MA, USA), 10 µM of Y27632 (Axon Medchem, Groningen, The Netherlands) and 10 ng/mL of TGF-β1.

### 2.5. Gene Expression Analysis

Gene expression analysis was used as an indicator of cellular differentiation. Briefly, both coated and non-coated wells were washed with 300 µL of PBS. Total RNA was isolated from each of the micromasses and dcECM using the Direct-zol RNA MiniPrep (Cambridge Biosciences, Cambridge, UK) according to the manufacturers’ instructions. Total RNA isolated was quantified using the Nanodrop 1000 spectrophotometer (ThermoFisher, Dartford, UK). Complementary DNA (cDNA) was synthesised by reverse transcription of 200 ng of total RNA using the high-capacity cDNA reverse transcription kit (Applied Biosciences, ThermoFisher, Dartford, UK). To detect messenger RNA (mRNA) transcripts, primers (exon spanning, designed using Primer3 Plus, NCBI or obtained from published sources) were used in conjunction with iTaq universal SYBR green supermix (Biorad, Herts, UK). Thermal cycling conditions were as follows: 10 min at 95 °C, with 40 cycles of 15 s at 95 °C, 30 s at 60 °C, and 20 s at 72 °C, on a Bio-Rad CFX1000 Real-Time System (Biorad, Herts, UK). Target gene quantification was achieved using the 2-ΔΔCT method described by Livak et al. [27], relative to *HPRT1* as the housekeeper gene.

### 2.6. Statistical Analysis

Data are expressed as the mean ± standard deviation (SD). Statistical significance was determined using one-way analysis of variance (ANOVA) with Bonferroni’s post-hoc corrections applied. Statistical significance is indicated on all graphs as follows: * *p* < 0.05, *** *p* < 0.001 (*n* = 3). All statistical analysis was performed using GraphPad Prism version 6.0 for windows (GraphPad Prism Software, La Jolla, CA, USA).

## 3. Results

### 3.1. dcECM Digests Can Coat Tissue Culture Plastic and Alternative Sterilisation Methods Elicit Different Surface Topologies

**Figure 1:**
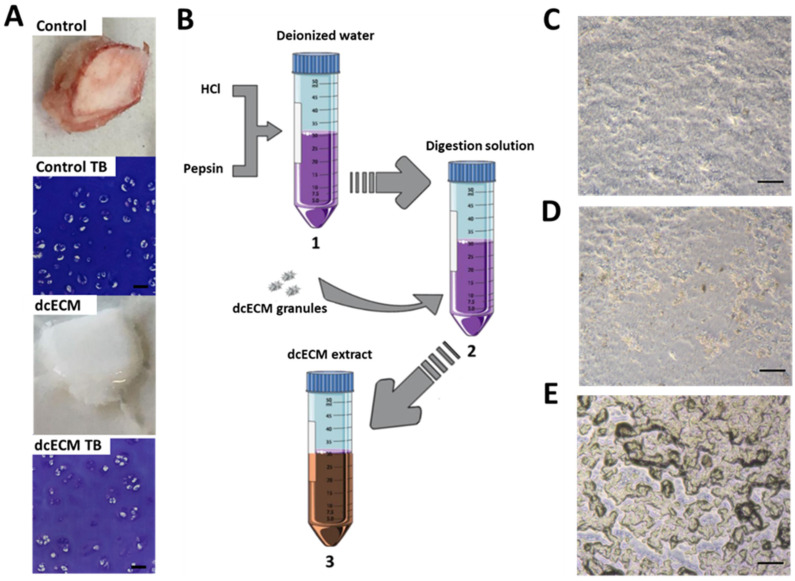
**Methodology to create dcECM extract and tissue culture surface coating.** (**A**) Representative macroscopic and Toluidine Blue (TB) image of control hypertrophic costal cartilage and dcECM (decellularised costal cartilage extracellular matrix); Scale Bar = 50 µm. (**B**) Methodology used to create dcECM extract: (1) Digestion solution (1 mg/mL Pepsin in 0.01 M HCL); (2) Sterile freeze-dried dcECM granules are digested at a concentration of 10 mg/mL; (3) extract after 96 h. (**C**) Brightfield images of tissue culture plastic coated with non-sterilised, (**D**) UV sterilised, and (**E**) autoclaved dcECM extract; Scale Bar = 100 µm.

Costal cartilage was decellularised to remove all cellular matter and preserve proteoglycan content as previously described, and as shown in Figure 1A. Following decellularisation, all remnants of blood and cellular material were removed, and the tissue was transformed to a brilliant white material. Despite this, Toluidine blue staining confirms the presence of glycosaminoglycans. The dcECM digests were processed as detailed in Figure 1B prior to post-process sterilisation, with the digests subsequently used to coat tissue culture plastic. Sterilisation with either UV (Figure 1D) or autoclave (Figure 1E) resulted in divergent surface topologies compared to non-sterilised coating (Figure 1C) when viewed by light microscopy. Autoclave-mediated sterilisation (Figure 1D) appeared to induce a surface that consisted of ‘islands’ of dcECM coating.

### 3.2. dcECM Surface Coatings Modify Chondrogenic Gene Expression from hPDCs and Promote Morphological Changes

**Figure 2:**
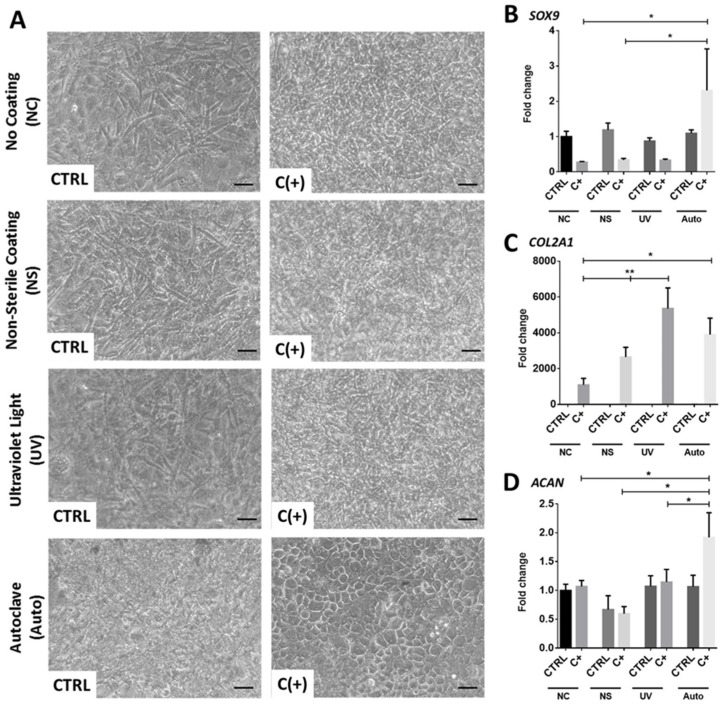
**Skeletal cell behavior on dcECM coatings.** (**A**) Brightfield microscopy images of hPDCs seeded on dcECM coatings that were either non-sterile (no post-processing sterilisation), UV sterilised, or autoclaved. The hPDCs were also seeded on non-coated tissue culture plastic. All samples were seeded in either CTRL or C(+) conditions and cultured for seven days. There was a distinct cobblestone pattern observed in hPDCs seeded onto the autoclaved dcECM coating (Scale bar = 50 µm). Chondrogenic gene expression of (**B**) *SOX9* (**C**) *COL2A1* and (**D**) *ACAN* from hPDCs seeded on non-coated surfaces or dcECM coatings under control conditions (CTRL) and C(+) chondrogenic conditions on non-coated surface (NC), non-sterile gel coating (NS), ultraviolet sterilised gel coating (UV) and autoclaved gel coating (Auto). (*n* = 3 * *p* < 0.05, ** *p* < 0.01). (Fold changes normalised to CTRL NC condition).

Next, dcECM-derived coatings were created, sterilised and tested for their ability to modify hPDC phenotype. Brightfield images taken at day 7 post-seeding highlighted distinct morphological changes in hPDCs that were seeded onto the autoclaved dcECM-derived (Auto) coating, reminiscent of a chondrocyte cobblestone pattern. Moreover, the differences observed in the Auto-coated well were limited to chondrogenic C(+) conditions; no other seeded group demonstrated any distinct morphological changes (Figure 2A). Gene expression at day 7 highlighted that only the Auto coating resulted in a significant two-fold upregulation in SRY-box transcription factor 9 (*SOX9)* expression (Figure 2B; *p* < 0.05) when compared to non-coated cultures in chondrogenic C(+) conditions. Interestingly, all coatings resulted in a significant upregulation of collagen type II alpha 1 chain (*COL2A1)* (Figure 2C) compared to noncoated plastic in C(+) (*p* < 0.05), whilst aggrecan (*ACAN)* (Figure 2D) was only upregulated in the Auto-coated plates in C(+) (1.7-fold vs. CTRL, *p* < 0.05). As such, the Auto coating was chosen as the optimal coating method, due to its ability to potently enhance chondrogenesis, and was subject to further optimisation and analysis.

### 3.3. High Density Culture of hPDCs Is Required for Chondrogenic Efficacy of dcECM (Auto)

**Figure 3:**
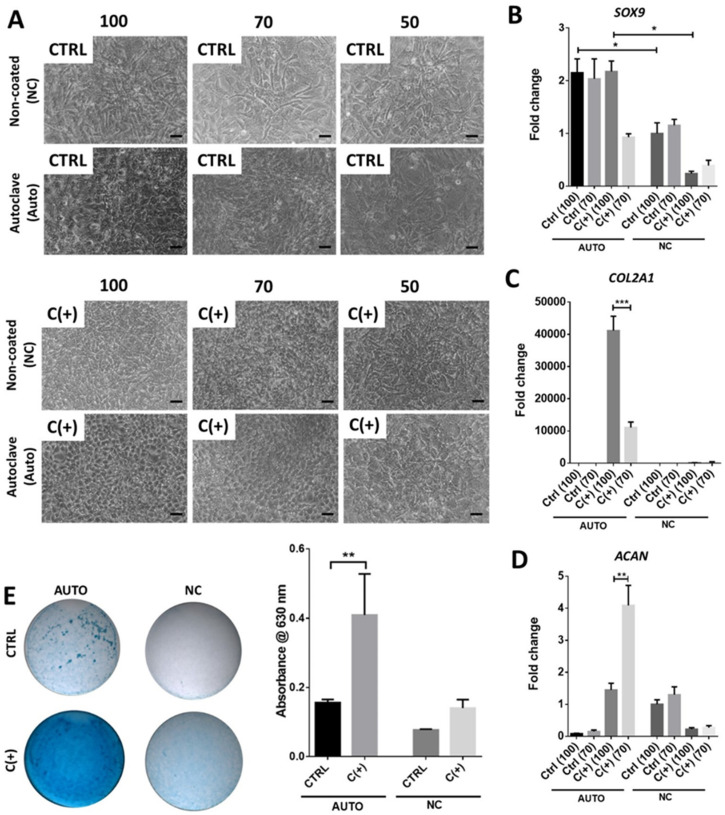
**Effect of cell seeding density on chondrogenic differentiation.** The hPDCs were seeded on the autoclaved dcECM extract coating (AUTO) and compared to cells on non-coated (NC) tissue culture plastic. The gel coating was air dried at 37 °C for 72 h before seeding. (**A**) Brightfield images were obtained seven days post-culture; Scale bar = 50 µm. Chondrogenic gene expression of (**B**) *SOX9* (**C**) *COL2A1* and (**D**) *ACAN*. The hPDCs were seeded in high density on non-coated surfaces or dcECM coatings under control conditions (CTRL) and chondrogenic C(+) conditions at densities of 100 × 10^3^ (100) 70 × 10^3^ (70) or 50 × 10^3^ (50) cells/well (96). (**E**) Brightfield microscopy images of Alcian blue (glycosaminoglycan) stained cultures and associated quantification. (*n* = 3 * *p* < 0.05, ** *p* < 0.01, *** *p* < 0.001).

To determine whether the apparent chondrogenic effect of the Auto coating was cell density dependent, three different cell densities were seeded onto Auto-coated plastic and imaged following seven days of culture. In confirmation of earlier results, the cultures seeded with 100,000 cells in C(+) conditions displayed a homogeneous cobblestone pattern which was progressively lost at the lower seeding densities of 70,000 and 50,000 cells. No distinct homogeneous patterns, or changes thereof, were noted in any other condition or at any other seeding density (Figure 3A).

Gene expression analysis revealed a significantly higher *SOX9* expression in Auto-coated plates (Figure 3B) than non-coated plates at a density of 100,000 cells irrespective of culture media (*p* < 0.05). Upon analysis of *COL2A1* expression, 100,000 cells cultured in C(+) conditions displayed a 3.7-fold increase over cultures with 70,000 cells (*p* < 0.001; Figure 3C). Negligible *COL2A1* expression was observed in other conditions. Interestingly, *ACAN* expression was significantly higher in C(+) conditions with 70,000 cells on Auto-coated surfaces (*p* < 0.01; Figure 3D). The enhanced chondrogenesis in the Auto-coated groups was further demonstrated by the increased Alcian blue staining at day 7 (100,000 cells) in C(+), indicative of increased sGAG deposition (Figure 3E). Furthermore, quantification of Alcian blue revealed that this increase was significant (*p* < 0.01)

### 3.4. Chondrogenic Effect Is Maintained over 21 Days, However, May Coincide with Hypertrophic Differentiation

**Figure 4:**
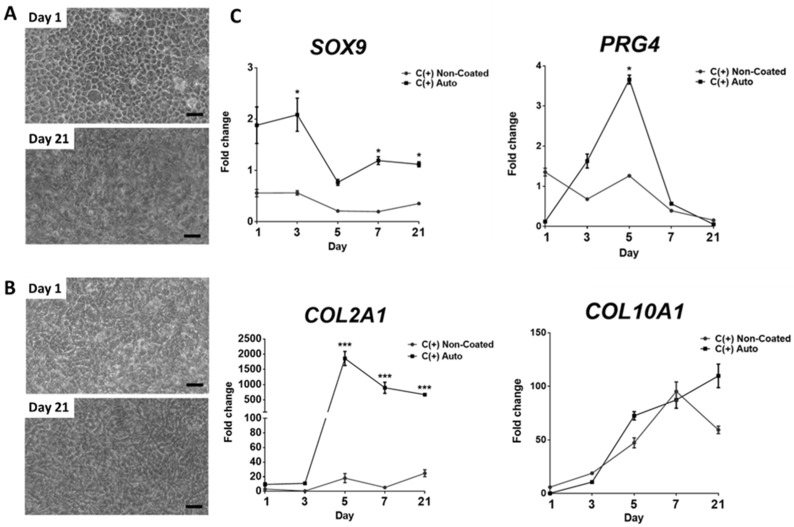
**Progression of cell phenotype and gene expression over time in response to dcECM (Auto) coating.** Brightfield microscopy images of hPDCs seeded at 100 × 10^3^ per well and cultured in C(+) on (**A**) autoclaved dcECM-extract coating and (**B**) non-coated tissue culture plastic at day 1 and 21. Scale bar = 50 µm. (**C**) Chondrogenic gene expression (*SOX9*, *PRG4*, *COL2A1* and *COL10A1*). Cells were cultured for a period of 1, 3, 5, 7 and 21 days in Chondrogenic C(+) conditions on non-coasted or dcECM (Auto) coating (*n* = 3 * *p* < 0.05, *** *p* < 0.001). (Fold changes normalised to cells in CTRL conditions). Statistical analysis was performed using one-way ANOVA corrected for multiple comparisons using Bonferroni’s post-hoc analysis.

To establish a detailed picture of the early changes in gene expression that underlie the enhanced chondrogenesis demonstrated by the Auto-coated tissue culture plastic, hPDCs were seeded onto Auto-coated and non-coated plates and monitored for up to 21 days in C(+) conditions. Brightfield microscopy imaging at day 1, revealed the establishment of a distinct cobblestone phenotype with hPDCs seeded onto Auto-coated samples in C(+), which was subsequently lost following 21 days in culture (Figure 4A). This pattern was not observed in non-coated cultures (Figure 4B). Upon analysis of longitudinal gene expression, a significant four-fold upregulation of the chondrogenic transcription factor *SOX9* was observed at day 3 when compared to non-coated conditions. The expression of *SOX9* in cells on Auto-coated surfaces remained higher than non-coated for the duration of the experiment (Figure 4C). Furthermore, there was a transient significant 2.7-fold (*p* < 0.05) upregulation in lubricin/proteoglycan 4 (*PRG4)* when Auto-coated cultures were compared to the non-coated controls at day 5. Expression of this marker returned to baseline by day 21 (Figure 4C). Upon analysis of *COL2A1* expression, there was a significant 100-fold (*p* < 0.01) upregulation compared to the non-coated condition at day 5. This significant increase vs. non-coated surfaces was maintained over the duration of the culture. Interestingly, both the Auto-coated show progressive increases in collagen type X alpha 1 chain (*COL10A1)* expression over the course of the experiment, with no significant difference between the conditions over the 21-day period (*p* > 0.01).

## 4. Discussion

With an ever-increasing demand for techniques to control cell behaviour in vitro, the ECM and its components are now being investigated to create novel methodologies to enhance cell isolation, survival, proliferation and differentiation. Herein, we describe the development of a method for creating dcECM-derived coatings that are capable of robustly enhancing chondrogenesis.

The dcECM-derived digests utilised in the current study were generated by pepsin digestion of freeze-dried porcine dcECM granules. This method was adapted from similar approaches to generate ECM-derived hydrogels [25,28,29]. Moreover, our studies suggest that the resultant ECM coating is capable of inducing chondrogenesis from human periosteal progenitor cells. A major challenge for the creation of such culture reagents is the formulation of a sterile extract that can be used in long term cultures. Despite the decellularisation process being conducted with sterile reagents, there remains a risk of bacterial or fungal contamination due to multiple steps within the procedure. Therefore, herein, the dcECM-derived coating was subject to further sterilisation using either UV or heat-mediated autoclave sterilisation. UV sterilisation is capable of inactivating vegetative bacteria and viruses, whilst also mediating the crosslinking of collagen components. Indeed, Rowland et al. suggested that cartilage-derived scaffolds that were crosslinked by UV exposure drive more robust chondrogenesis and sGAG deposition when seeded with MSCs [30]. As such, this sterilisation methodology may have beneficial effects beyond sterilisation. Conversely, UV is ineffective at inactivating bacterial spores, mycobacteria and prions, and therefore has to be used with caution if considering future clinical applications [31]. There are no known studies that investigate the use of an autoclave for heat-mediated sterilisation of the ECM-derived products, potentially due to the high temperatures that could result in heat-induced conformational changes/denaturation of protein components of the ECM. Despite the potential destructive effect of heat on the ECM, autoclave sterilisation has a proven track record of being able to eliminate all microbiological contamination including spores.

Post-sterilisation, the dcECM digest was used to coat tissue culture plastic through drying, which was employed in an attempt to concentrate the ECM components onto the culture surface. A similar approach was proposed by DeQuach et al. who used pepsin digested decellularised cardiac tissue-derived coatings and reported efficient ECM deposition onto tissue culture plastic when allowed to dry [23]. Interestingly, each sterilisation methodology resulted in a unique surface topology post-coating, which may be the result of differing levels of crosslinking or protein denaturation. Brightfield images of the cells seeded onto the autoclaved (Auto) dcECM-digest coated surface remarkably revealed a cobblestone morphology, as early as day 1 in C(+) conditions. The rounded cobblestone morphology is a distinct characteristic of chondrocytes seeded onto tissue culture plastic before dedifferentiation [32]. Moreover, the rounded morphology has also been linked with the upregulation of chondrogenic genes and, subsequently, cartilage-specific matrix deposition [9,10]. These findings were confirmed by assessing the associated gene expression data. The hPDCs seeded onto Auto samples in C(+) conditions demonstrated significantly higher *SOX9* expression when compared to the non-coated control conditions; however, with no significant difference vs. CTRL media. This may indicate that the Auto dcECM coating is promoting *SOX9* expression in an autonomous manner. The upregulation of *SOX9* could be a direct result of growth factors sequestered in the dcECM and/or the creation of a chondroinductive topography as a result of the Auto coating process. Indeed, previous studies have highlighted the importance of nanotopography on cellular morphology and chondrogenesis [8]. Upon analysis of *COL2A1* expression, it is clear that C(+) chondrogenic media is required for its expression, which may be a direct result of TGFβ. Indeed, this growth factor has been associated with increased *COL2A1* expression in multiple studies [33,34].

Cell–cell interactions mediate chondrogenesis [35]. Therefore, initial cell seeding density has a significant impact on chondrogenic differentiation, especially with regards to mesenchymal stromal cell (MSC) populations [36]. Interestingly, the distinct rounded morphology cobblestone pattern observed with Auto-coated plates in C(+) conditions was the most distinct at 100,000 cells, and was progressively lost at lower densities. These data are further supported by gene expression analysis, which reveals a significantly higher expression in the chondrogenic marker *COL2A1* in chondrogenic conditions that contained cells seeded at a density of 100,000 compared to 70,000. Interestingly, the opposite was true for *ACAN* expression, whilst again *SOX9* expression appeared to be independent of seeding density or media conditions. This may indicate that alternative terminal chondrocyte phenotypes may promoted by different seeding densities, or differentiation was occurring at different rates in each culture.

With this in mind, it was essential to investigate earlier and later time points to monitor changes in gene expression using well validated markers of cell phenotype. As such, gene expression from the Auto-coated group was compared to non-coated controls in chondrogenic C(+) conditions. Interestingly, the expression of *SOX9*, *PRG4*, *COL2A1* and *COL10A1* indicated that hPDCs may be transitioning through distinct chondrocyte phenotypes. Indeed, day 1–7 cultures may represent an articular cartilage phenotype, whilst D7–21 represents transition to a hypertrophic phenotype. This speculation can be justified due to *SOX9* having a critical role in promoting the expression of cartilage matrix-associated genes [37]; whilst the transient expression of *PRG4* expression suggests increased abundance of lubricin, a key component for the lubrication of the articular joint. Interestingly, higher lubricin expression has been noted in chondrocytes that are cultured from healthy human articular when compared to articular chondrocytes extracted from patients with osteoarthritis [38]. As such, the ability of the Auto coating to drive the upregulation of *PRG4* may allow for the creation of novel expansion techniques for chondrocytes for use in ACI. Moreover, significantly higher *COL2A1* expression is maintained through to 21 days, again indicating the differentiation towards a stable chondrogenic phenotype. Despite this, a progressive increase in the marker *COL10A1* may suggest the gradual differentiation towards a pre-hypertrophic phenotype [39]. Recently, Shi and colleagues indicated that addition of C-type natriuretic peptide (CNP) combined into the culture conditions of MSC-derived chondrocytes to maintain a stable chondrocyte phenotype and prevent hypertrophy [40]. Future work may benefit from investigating other factors that could be used in combination with the Auto coating to preserve a stable chondrogenic phenotype.

Overall, these data highlight the capacity of the Auto dcECM-derived coating to drive rapid early chondrogenesis. Although it was surprising that the autoclave sterilisation method resulted in a highly active coating, it should be noted that high heat is used for the industrial generation of gelatin, which is also routinely used for the cell culture coatings. Finally, although the prochondrogenic efficacy of Auto coating is unquestionable, the mechanisms by which it interacts with hPDCs remains unexplored. Indeed, the Auto dcECM-derived coating may result in the formation of distinct nano-topographical features present on the coated surface, and these features may subsequently drive a transcriptional change in the attached cells. Future work should therefore look to further define changes in surface topography using scanning electron microscopy or atomic force microscopy.

In conclusion, the use of Auto dcECM-coated plastic is a promising approach for the rapid differentiation of hPDCs, potentially shortening the time required for their chondrogenic priming. Moreover, the speed of differentiation was vastly improved over traditional methods for the differentiation of MSCs, which can take around 2–3 weeks [36]. This study may provide the basis for the adoption of a new cell culture coating based on decellularised hyaline cartilage with the unique ability to rapidly differentiate progenitors into chondrocytes. This is likely to have far reaching applications within cell biology and regenerative medicine fields.

## Data Availability

The data presented in this study are available on request from the corresponding author.
